# Graphene Oxide Papers in Nanogenerators for Self-Powered Humidity Sensing by Finger Tapping

**DOI:** 10.1038/s41598-020-64490-7

**Published:** 2020-04-30

**Authors:** Faezeh Ejehi, Raheleh Mohammadpour, Elham Asadian, Pezhman Sasanpour, Somayeh Fardindoost, Omid Akhavan

**Affiliations:** 1 Institute for Nanoscience and Nanotechnology, Sharif University of Technology, Tehran, 14588-89694 Iran; 2 Department of Medical Physics and Biomedical Engineering, School of Medicine, Shahid Beheshti University of Medical Sciences, Tehran, Iran; 3 School of Nanoscience, Institute for Research in Fundamental Sciences (IPM), P. O. Box, 19395-5531 Tehran, Iran; 4 Department of Physics, Sharif University of Technology, Tehran, 11155-9161 Iran

**Keywords:** Devices for energy harvesting, Sensors and biosensors

## Abstract

Triboelectric nanogenerators (TENGs) offer an emerging market of self-sufficient power sources, converting the mechanical energy of the environment to electricity. Recently reported high power densities for the TENGs provide new applications opportunities, such as self-powered sensors. Here in this research, a flexible graphene oxide (GO) paper was fabricated through a straightforward method and utilized as the electrode of TENGs. Outstanding power density as high as 1.3 W.m^−2^, an open-circuit voltage up to 870 V, and a current density of 1.4 µA.cm^−2^ has been extracted in vertical contact-separation mode. The all-flexible TENG has been employed as a self-powered humidity sensor to investigate the effect of raising humidity on the output voltage and current by applying mechanical agitation in two forms of using a tapping device and finger tapping. Due to the presence of superficial functional groups on the GO paper, water molecules are inclined to be adsorbed, resulting in a considerable reduction in both generated voltage (from 144 V to 14 V) and current (from 23 µA to 3.7 µA) within the range of relative humidity of 20% to 99%. These results provide a promising applicability of the first suggested sensitive self-powered GO TENG humidity sensor in portable/wearable electronics.

## Introduction

Energy harvesting is an area of tremendous attention because of the huge worldwide energy demands motivating considerable research on self-powered and autonomous systems^1^. In the last decade, nanogenerators have been introduced as sustainable self-sufficient power sources, converting energy from the environment into electrical energy^2^. They can be categorized to piezoelectric^3^, triboelectric^4^, and pyroelectric^5^ nanogenerators. Triboelectric nanogenerator (TENG) works on the basis of accumulation of electrostatic charges produced on the surface of two dissimilar materials while they are brought into a physical contact^4^. When the surfaces are separated by a mechanical force, the induced triboelectric charges generate a potential drop, leading to produce electric current^6^. Since their invention in 2012^7^, TENGs have shown a rapid development in diversity of structures, amount of output power, and range of application, such as self-powered sensors^8,9^, biomedical monitoring^10^, and electronic skin^11^.

Among the various energy-related materials, graphene-based materials have been studied extensively in the field of energy harvesting in recent years^12^. It has been demonstrated that the reactive oxygen functional groups of graphene oxide (GO), beside its high specific surface, makes it a good candidate as an energy-related material^13^. Moreover, high intrinsic mechanical strength of GO provides the ability of producing a flexible and stable thin layer, which let the induced charges achieve to the back contact in TENGs. Tian and his co-workers reported a flexible electrostatic nanogenerator based on GO film, with a multilayer structure of Al/PI/GO/PI/ITO with the output power of 60 nW^14^. The fabricated GO-TENG was able to generate a peak voltage of 2 V and a current of 30 nA upon the applying of a 15 N force at a rate of 1 Hz. Very recently, Wang’s group proposed a GO single-electrode-based TENG with maximum power of 5 mW (power density = 3.13 W/m^2^) at 3 Hz^15^. The short circuit current (I_sc_) of 55 μA and the open-circuit voltage (V_oc_) of 1100 V were obtained with latex-gloved hand patting. Moreover, the dynamic force sensing and the sterilizing performance of the aforementioned TENG were investigated. There are also several researches on designing novel TENGs containing GO which demonstrate weaker results and utilize more sophisticated fabrication processes^16,17^.

Beside energy harvesting field, detecting humidity is from promising applications of GO because of the strong interactions between water molecules and oxygen functional groups on the surface^18^. Humidity sensing plays a key role in domestic control, medical care, agricultural moisture monitoring, and industrial applications^19^. GO-based humidity sensors have been widely under attention due to notable properties such as simple, low-cost, and large-scale preparation of GO material along with high proton-conductivity in exposure to water molecules^20^. GO humidity sensors operate on the basis of detecting the variation of impedance or capacitance due to the inclination of the water molecules to adsorb on the surface of GO^21–26^. Both types possess the ability of fast response humidity sensing with high sensitivity in a wide range of relative humidity (RH). Impedance sensors detect a relatively low humidity over a wide frequency range, but generally require professional high-performance impedance spectroscopy. On the other hand, the capacitive sensors detect the humidity by measuring the capacitance change which consists of the interdigitated conductive electrodes and GO-based sensing materials as the dielectric connected to the LCR meter. For practical usage of a humidity sensor, not only the sensing performance such as fast response and high sensitivity, but also low cost, easy fabrication, facile integration, and good flexibility are significant factors, which are generally depends on the sensing materials and fabrication methods^20^. In spite of various investigations demonstrated the effect of utilization of polymers^27,28^, metal oxides^29–31^ and 2D materials^32,33^ alongside GO, with the purpose of enhancement of humidity sensing, the fabrication method has become relatively more sophisticated. Moreover, the recent development of the Internet of Things (IoT) provides a progressive research field on integrating numerous sensors, which demands a new approach to the power supply issue^19^.

In order to eliminate the drawbacks of utilizing the batteries as power sources in sensors (specifically cost and environment issues), considerable investigations have been launched into the fabrication of self-powered sensors^2^. In spite of the fact that TENGs are considered as a promising option for self-powered sensors^2^, limited numbers of self-powered humidity sensors based on TENGs have been reported^34–36^. In the reported TENG-based humidity sensors, the TENG is generally used as an external power source connected to a humidity-sensitive electrode, analogous to a resistive sensor. In spite of being battery-free, there are some limitations in utilizing these sensors, such as low sensitivity and confined range of RH^37–39^. Moreover, the only value which demonstrates the amount of RH is the output voltage and no current variation was observed^35^. Beside the sensing properties, flexibility is a key factor for utilizing a sensor in wearable electronics, which was not considered in many previous studies of TENG-based humidity sensors. Therefore, the direct implementation of a humidity sensitive flexible material as one of the TENG electrodes may offer a self-powered humidity sensor with a less complicated structure as well as higher sensitivity and wider range of detection.

In this research, a sustainable GO-based TENG has been introduced, which shows superior power density and high sensitivity of humidity sensing, in comparison with the previous studies. Using the modified Hummer’s method, as well as drop casting technique for the synthesis of GO sheets and construction of the electrode, the whole procedure of production is extremely straightforward. Besides, utilizing GO paper and Kapton film as the electrodes, makes the GO TENG an appropriate candidate for wearable electronics, due to its flexibility and low weight. Applying the as-fabricated GO TENG as a self-powered humidity sensor is reported for the first time in this paper. Here, the GO electrode directly senses the amount of relative humidity, while corporates in signal generation as well. The results demonstrate considerable variation in the amount of both voltage and current at different values of RH. Investigations of humidity sensing were performed on large and small sizes of the electrodes, which present the fabricated TENG as an applicable option for portable devices, as well as industrial implementations. Therefore, our suggested GO TENG provides flexibility and high sensitivity of dual sensing in a wide range of RH and can be conveniently applied as a routine battery-free humidity sensor in desired places.

## Results and Discussion

**Figure 1:**
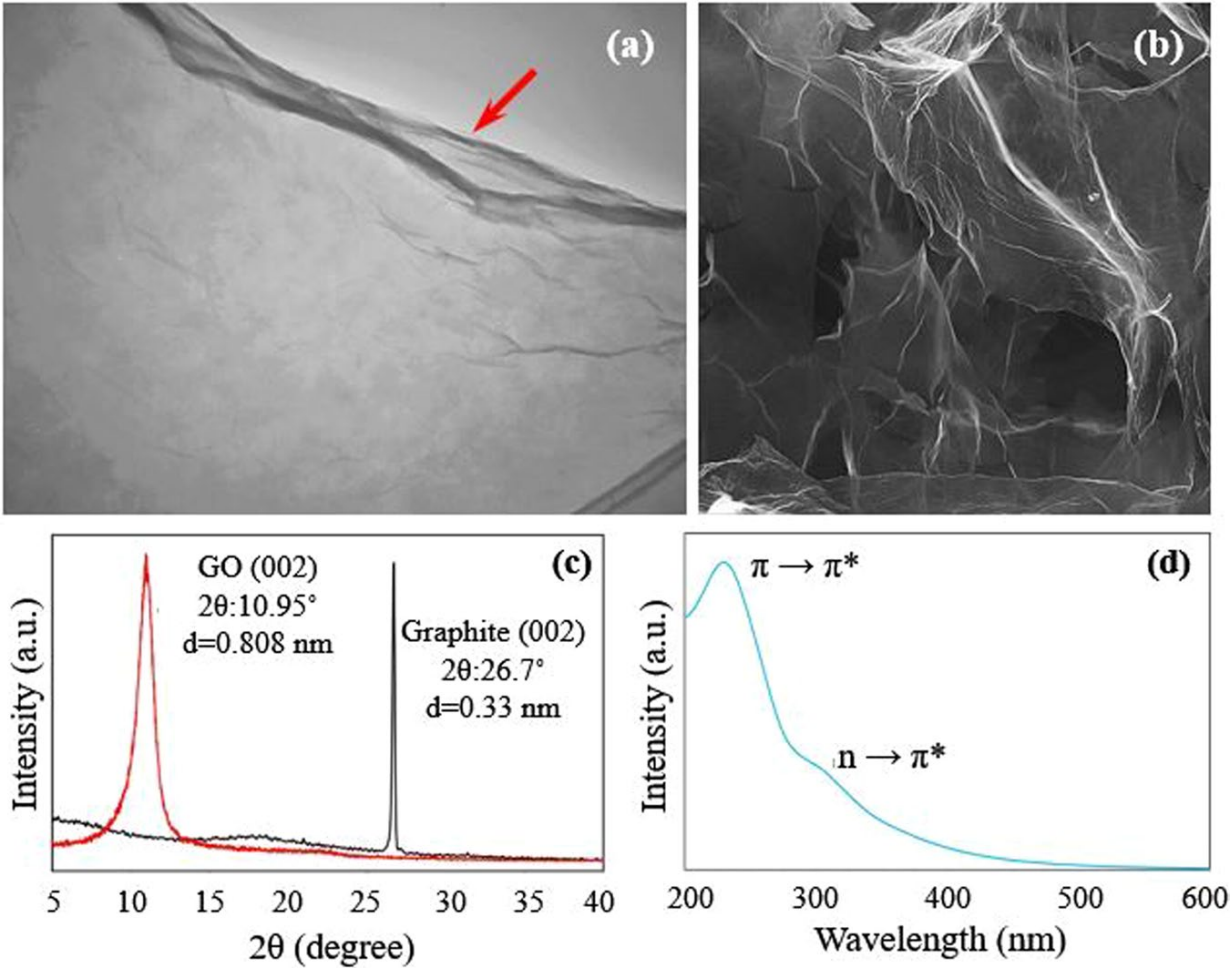
Characterization of GO suspension. (**a**) TEM and (**b**) SEM images of the prepared GO nanosheets. (**c**) XRD pattern of GO nanosheets compared to primary graphite powder. (**d**) UV-Vis absorption spectrum of GO sheets.

The SEM and TEM images of the produced GO suspension (Fig. 1a,b), obviously show the ripples and wrinkles (see the red arrow) of graphene sheets. The intense (002) peak in the XRD pattern of the GO nanosheets compared to the primary graphite powder indicates the effective exfoliation of carbon sheets (Fig. 1c). These results are consistent with the corresponding UV-Vis absorption spectrum of GO sheets (Fig. 1d). The peak at 230 nm is related to π-π* transitions of the aromatic C–C bond and the shoulder around 300 nm corresponds to n-π* transition of the C=O bond transition.

**Figure 2:**
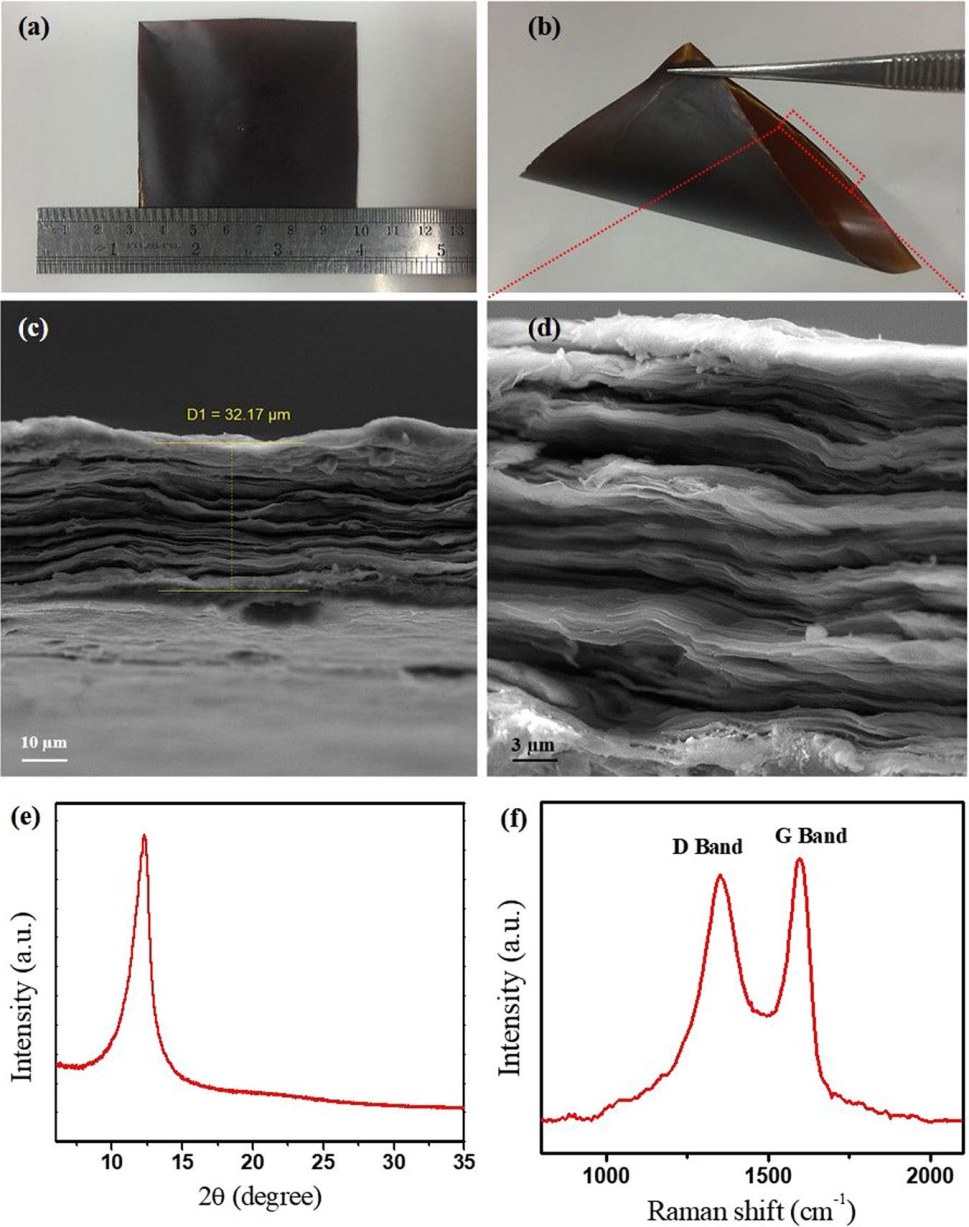
Characterization of the prepared free-standing GO paper. (**a**,**b**) Digital photograph of the GO paper. (**c**) Cross section SEM image. (**d**) SEM image with higher resolution. (**e**) XRD pattern. (**f**) Raman spectrum.

The digital photograph of free-standing graphene oxide paper fabricated by drop casting of graphene oxide solution is shown in Fig. 2a. Since the GO paper is absolutely free-standing and almost flexible (Fig. 2b), it is appropriate for a great number of applications, such as wearable nanogenerators^40^ as well as various kinds of sensors. The thickness of the layer is obtained to be around 32 µm (Fig. 2c), while its surface area is 50 cm^2^ approximately (Fig. 2a). Figure 2d demonstrates the SEM image of the stacked GO sheets simply formed the GO paper after drying in the air. In the X-Ray diffraction pattern obtained from the prepared GO paper, a sharp peak was observed at 12.3° which confirms that the layer consists of exfoliated GO sheets (Fig. 2e) and no stacking was occurred. Moreover, the Raman spectrum consists of characteristic D and G-band of GO sheets appeared at 1351 and 1593 cm^−1^, respectively (Fig. 2f). These observations together confirmed the formation of a free-standing GO paper composed of individual sheets.

**Figure 3:**
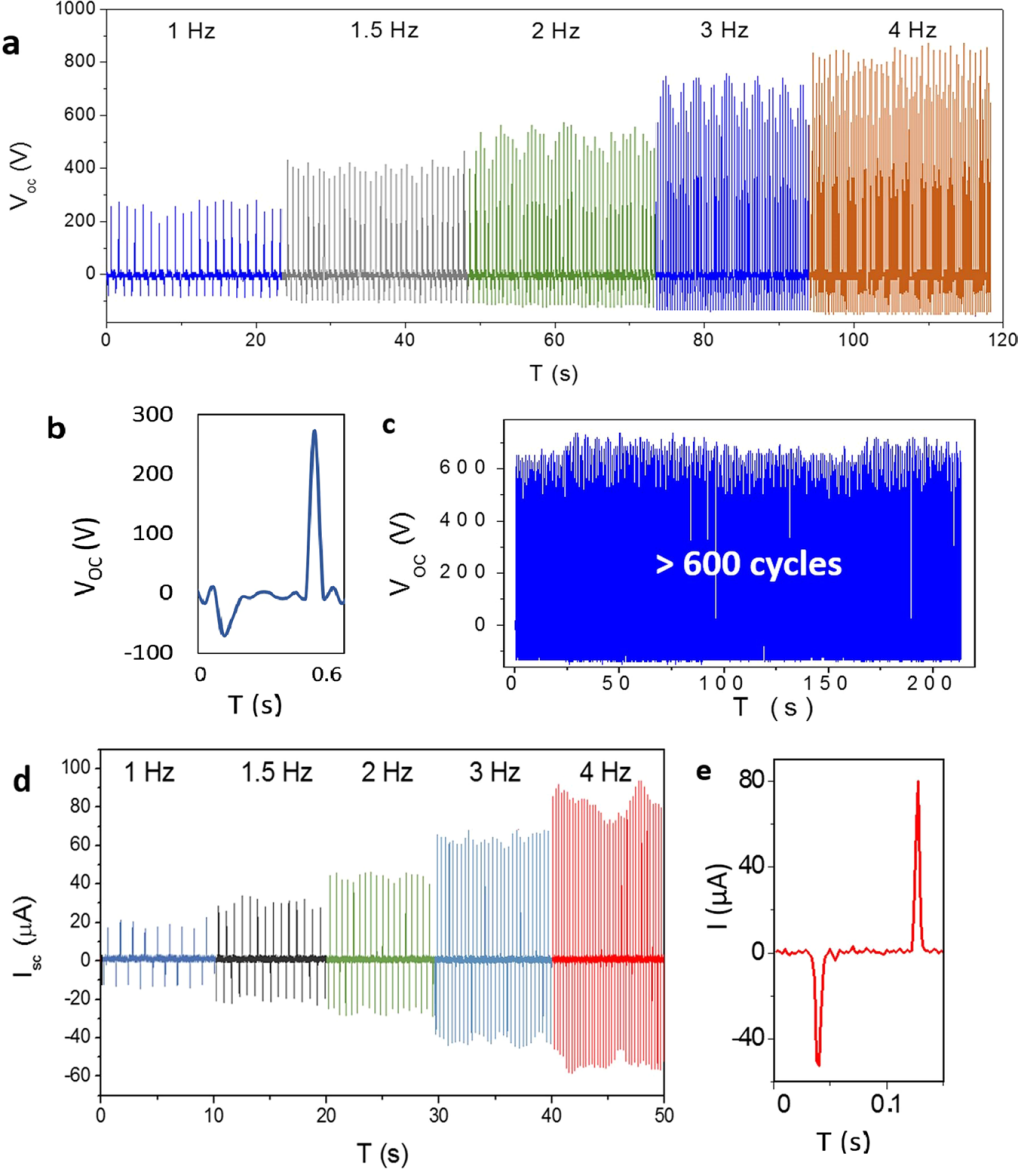
Output signals of the fabricated GO TENG. (**a**) Open-circuit voltage under frequencies from 1 to 4 Hz. (**b**) Magnified voltage peak at frequency of 1 Hz. (**c**) Stability of the output voltage at 3 Hz. (**d**) Short-circuit current under frequencies from 1 to 4 Hz. (**e**) Individual current peak at 4 Hz.

Figure 3a demonstrates the AC voltage produced by GO TENG under various quantity of tapping frequency in vertical contact-separation mode. In the open-circuit condition, the measured voltage at 1 Hz shows an average value of 282 V (Fig. 3b) which is reached to maximum value of 870 volt at 4 Hz. The alteration of the maximum peak value at higher rates intensified, which may occur due to the higher accumulation of surface charges, as well as less time for charge transfer. The stability of the generated voltage has been confirmed in Fig. 3c for over 600 cycles of tapping.

The short-circuit current demonstrates more outstanding results in Fig. 3d. The value of the current at 1 Hz is 20 µA, reaching a maximum value of 90 µA at 4 Hz, which can be easily amplified up to 1.7 mA using a transformer (Fig. S1). As mentioned for voltage output, the variation of maximum current peak is intensified at higher frequencies, however, the time behavior of individual peak maintains its primary form (Fig. 3e). In order to utilize the produced electrical current as a power source in a self-powered device, it should be rectified by a full wave rectifier through diode bridge (Fig. S2).

**Figure 4:**
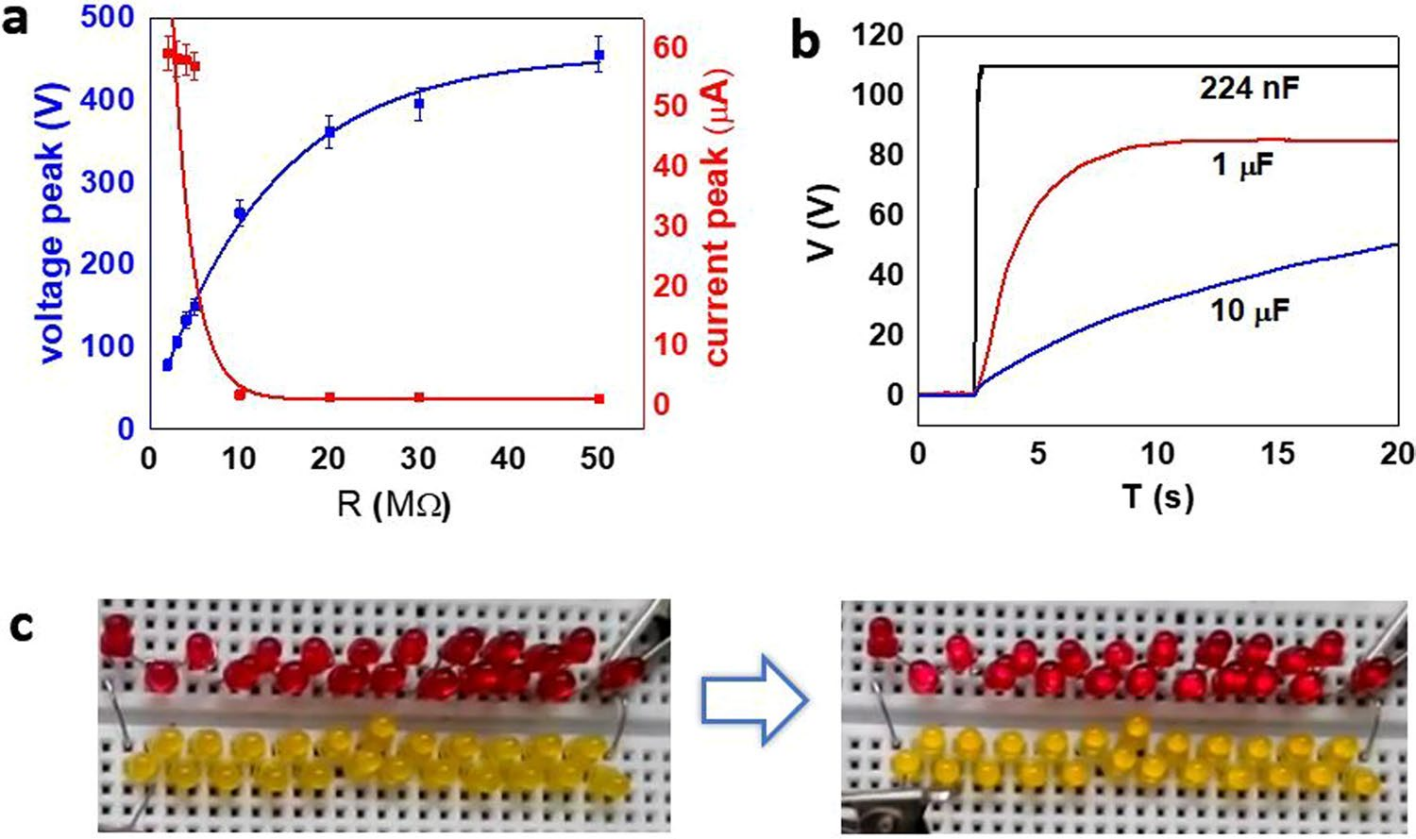
Characterization of the GO TENG. (**a**) Dependence of the voltage and current peak on the external loading resistance. (**b**) Charging of capacitors with three different values of capacitance. (**c**) Lightening of 44 diodes by assistance of the GO TENG.

Generally, the effective power of a TENG is dependent on the match with the loading resistance^15^. In order to calculate the maximum power, and subsequently power density, the quantities of voltage and current were measured for different amount of resistance (Fig. 4a). By increasing the resistance, the average of current peaks drops down to near zero, while the voltage peaks elevate up to the open-circuit value. The maximum power at resistance of 5 MΩ was 8.49 ± 0.67 mW (Fig. S3), which corresponds to the power density of ~1.3 W/m^2^ at 2 Hz. Such a superior performance provides an extensive range of applications for the fabricated GO TENG. Moreover, the TENG is able to charge a 224 nF and a 1 µF capacitor in less than 1 and 7 seconds, respectively (Fig. 4b). Under tapping at 3 Hz, our TENG can light up 44 light-emitting diodes (LEDs), as shown in Fig. 4c and movie 1 (see the Supporting Information).

**Figure 5:**
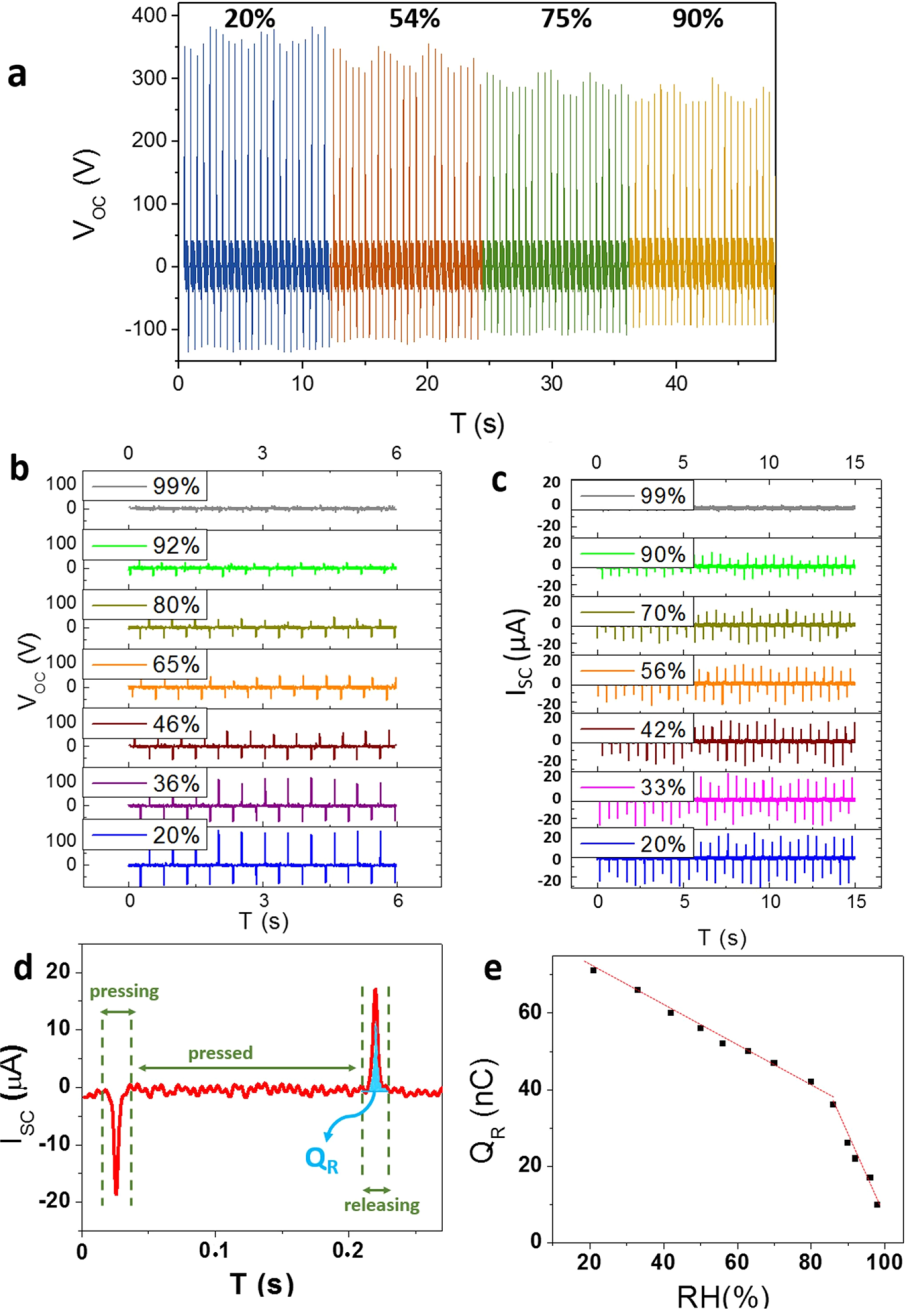
Electrical outputs under different values of RH. (**a**) Variation of the open-circuit voltage for large electrode. (**b**) Variation of the generated voltage and (**c**) current for small electrode. (**d**) The peaks of pressing and releasing in a single tapping. (**e**) The amount of transferred charge versus RH during releasing for the medium current peak.

Since humidity sensing is a conventional application of GO, we investigated the impact of variation of ambient humidity on the generated voltage and current. Primary humidity sensing tests were performed on the aforementioned GO TENG at the frequency of 2 Hz. By increasing the RH, the voltage generated by the TENG reduced gradually (Fig. 5a). It can be expressed that elevating the percentage of relative humidity (%RH) leads to increase the amount of adsorbed water molecules on the surface of GO, which results in diminishing the superficial induced charges via tapping. The variation of output current can be observed in Fig. S4.

Decreasing the size of the electrode from 8 × 8 cm^2^ to 2 × 2 cm^2^ led to a more considerable reduction in both voltage and current output, which is depicted in Fig. 5b,c. By increasing the relative humidity from 20% up to 99%, the open-circuit voltage decreased from 144 V to 14 V and the short-circuit current reduced from 23 µA to 3.7 µA. Enhancement of the sensing results is due to the surface area of the applied electrode: during the tapping process, the molecules which interact physically with the surface may desorb and be driven out of the space between the two electrodes. In order to sense the value of RH more accurately, the molecules should move between the electrodes during the tapping and physisorb to the surface of the electrode. At larger sizes, this movement is restricted to the edges and hardly reaches to the center of the electrode. At smaller sizes, diffusion of molecules through the space between the electrodes is more facile, which leads to higher adsorption on the surface and more efficient reduction of the surface charge.

According to the equation of I.dt = dQ, the amount of surface charge can be obtained via integrating the current peak over time. Figure 5d demonstrates the whole process of press-release for a single tapping. The surfaces under the peaks of pressing and releasing refer to the charges transferred between two electrodes^41^. By increasing the humidity, the amount of transferred charge should decrease. According to the Fig. 5e, by increasing the RH, the transferred charge corresponding to the medium releasing peak gradually diminishes from 71 nC to 10 nC. It should be mentioned that two different paces of reduction can be observed before and after RH = 85%.

**Figure 6:**
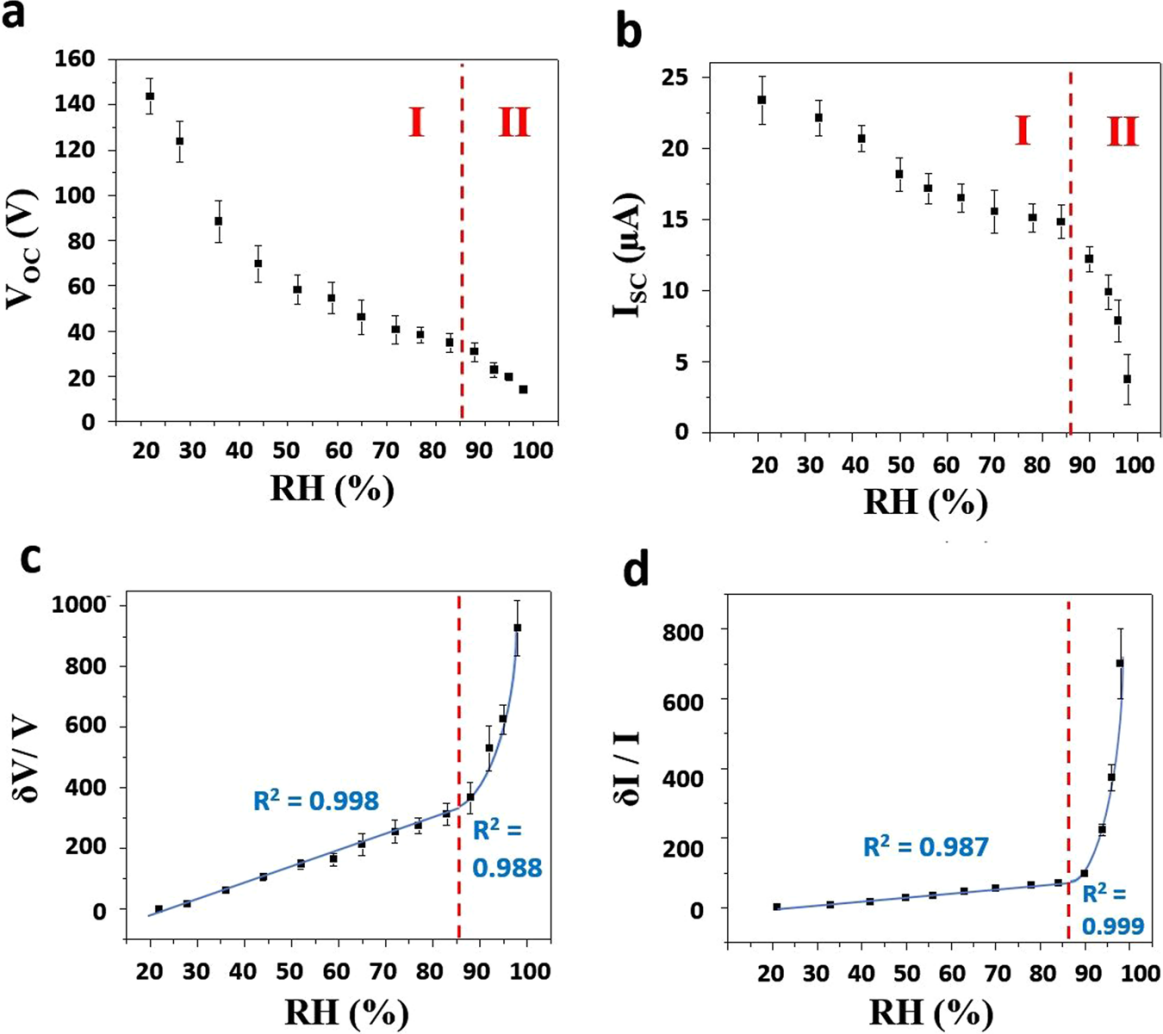
Sensing diagrams. Variation of the open-circuit voltage (**a**) and the short-circuit current (**b**) versus RH. The response voltage (**c**) and current (**d**) diagrams for different amounts of RH.

**Figure 7:**
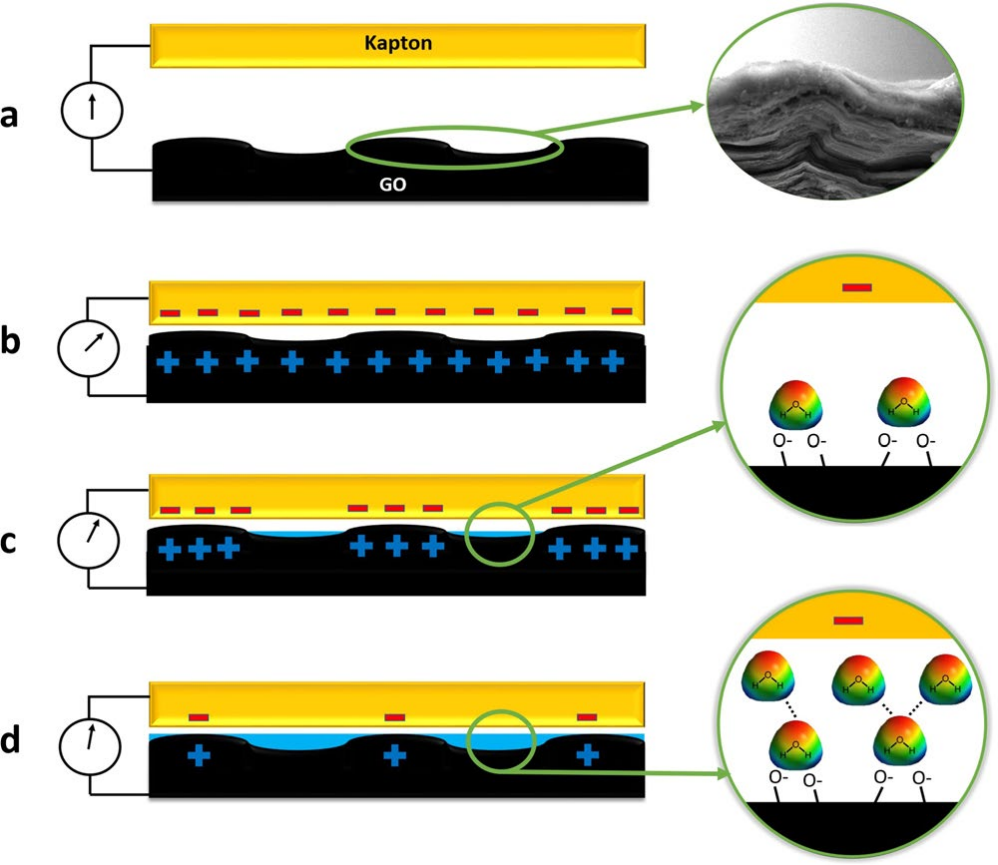
Sensing mechanism. (**a**) Schematic of the fabricated GO TENG. Inset: SEM image of the surface of GO paper. (**b**) Electrical current generation between electrodes. (**c**) Reducing the current in the presence of medium humidity. Inset: adsorbed water molecules through double hydrogen bonding. (**d**) Minimizing the current amount under high humidity and formation of a continuous water layer. Inset: adsorbed water molecules through single hydrogen bonding.

Figure 6a,b depict a similar two-step behavior in decrease of voltage and current around RH = 85%. In order to investigate the obtained data thoroughly, the response diagrams should be plotted. Herein, the response value for voltage (and also similarly for current) is defined as: (V_0_-V)/V, while V_0_ and V correspond to the voltage value at RH = 20% and the desired RH, respectively. Both of the response diagrams (Fig. 6c,d) show an elevating slope by increasing the RH. The response value of generated voltage reached to 930%, while the maximum value for current response is about 700% at RH = 99%. Here, the slope generally refers to the sensitivity of the sensor. The two- step behavior of the sensor (linear increase before RH = 85% and then exponential rise after RH = 85%) reveal the corresponding mechanism of humidity sensing, which is displayed in Fig. 7.

In general, the response of the GO TENG sensor to ambient humidity relates to the physical adsorption of water molecules on the surface of GO layer (Fig. 7a). In order to interpret the relation between the output current and RH, the following adsorption process of water molecules on GO surface can be suggested (Fig. 7b-d).

At low RH, water molecules are primarily physisorbed and condensed onto the available active sites (including hydrophilic groups and vacancies) of the GO surface through double hydrogen bonding^42^. In such circumstances, the water molecules form an obstacle to electrostatically induced charges between the induced negative charges on the Kapton and the induced positive charges on the GO film via reducing the contact surface, resulting in the formation of a depletion region. Therefore, this phenomenon causes a decrease in the output current of the GO TENG at low RH regime (Fig. 7c).

As the RH increases, water molecules are adsorbed physically on the hydroxyl groups of the first physisorbed layer through single hydrogen bonding (Fig. 7d). Simultaneously, the permeation of water molecules into the internal layers of GO may occur. Thereafter, the water molecules become identical to those in the bulk liquid and forming a continuous layer^21^. In that case, a continuous water layer creates a uniform barrier layer for induction of positive charges on the GO surface, which results in the formation of a continuous depletion region and would lead to a more rapid decrease of current at higher RH. Calculated amount of the induced charge on the surface under various RH (Fig. 5d) confirms the above mechanism.

**Figure 8:**
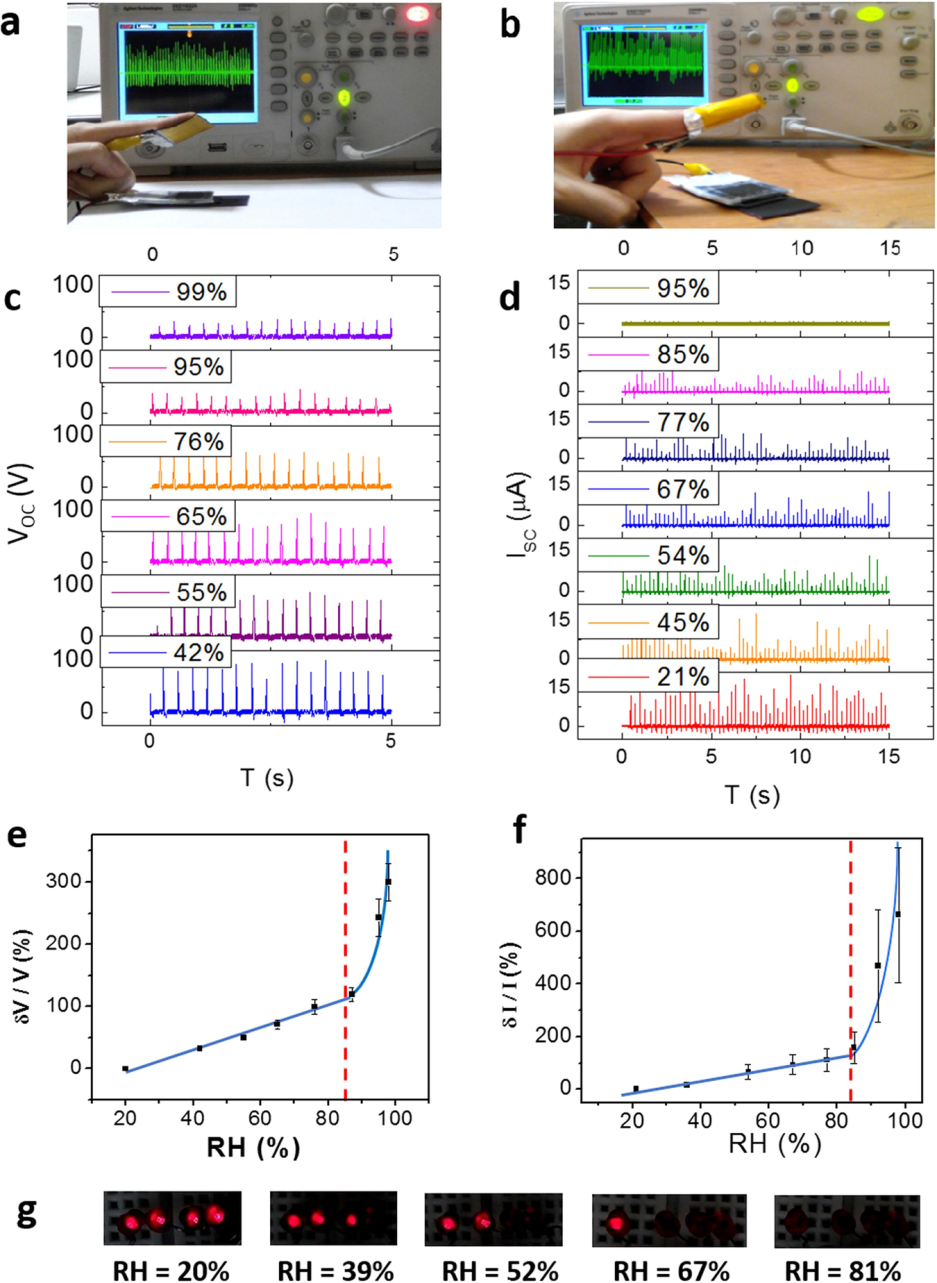
Humidity sensing under finger tapping. (**a**) Top electrode connected to a finger. (**b**) Top electrode wrapped around a finger. Variation of the open-circuit voltage (**c**) and the short-circuit current (**d**) at various amounts of RH. The response value of the output voltage (**e**) and current (**f**) versus RH. (g) The number of LEDs which can be lightened by GO TENG at different values of RH.

In order to investigate the performance of the fabricated GO TENG as a self-powered humidity sensor, the sensing tests were repeated under uniformly finger tapping. In this case, the Kapton electrode was connected to (Fig. 8a) or wrapped around (Fig. 8b) the finger and tapped on the GO electrode, as shown in movie 2 (see the Supporting Information). Figure 8c,d demonstrate the output voltage and current, respectively. As it is observed, the generated voltage and current peaks are not as uniform as before, especially for current diagrams. Figure 8e,f show that the behavior under finger tapping condition is generally similar to the aforementioned results of Fig. 6c,d. In spite of the large quantity of standard deviation for current response, it is obvious that the total amount of generated current during 15 seconds decreased by elevating the RH, according to the Fig. 8d. In other words, measuring the produced power by the GO TENG via finger tapping, would be a high reliable method to indicate the amount of ambient humidity. As an evidence, the electrical power generated at various amounts of RH leads to lighten different numbers of LED, as shown in Fig. 8g. Table 1 summarizes the performance of the flexible self-powered GO TENG humidity sensor in comparison with the other humidity sensors based on GO (not self-powered) or TENGs (not utilizing GO as sensing material).

**Table 1:** Comparison of the sensing performance of the self-powered GO TENG humidity sensor with other humidity sensors based on GO or TENG.

Type	Sensing material	Flexible/Rigid	Response time	Humidity range	Long-term stability	Ref.
Capacitive	GO	Rigid	10.5 s	15–95%	30 days	^21^
Impedance	GO	Flexible	30 ms	10–90%	72 h	^22^
Resistive	GO	Flexible	100 s	35–80%	—	^23^
Capacitive	GO	Rigid	<0.1 s	12–97%	—	^25^
TENG	PTFE	Rigid	—	20–100%	—	^34^
TENG	RGO/PVP	Rigid	2.8 s	23–97%	>1 month	^35^
TENG	PFSA*	Flexible	30 s	25–95%	—	^36^
TENG	GQD	Rigid	—	30–70%	—	^43^
TENG	GO paper	Flexible	~5 s	20–99%	>8 months	This work

*Perfluorosulfonic acid ionomer.

In summary, a sustainable GO-based TENG which shows superior power density (1.3 W/m^2^) has been fabricated, through a straightforward method. The outstanding I_sc_ and V_oc_ with the values of 90 µA and 870 V, respectively, at a frequency of 4 Hz are obtained. Applying the fabricated TENG as a self-powered humidity sensor demonstrates promising results. Increasing the relative humidity of the environment, gradually reduced the generated voltage from 144 V at RH = 20% to about 14 V at RH = 99%, while short-circuit current reduced from 23 to 3.7 µA. Performing sensing tests under finger tapping presents a lightweight self-powered humidity sensor. This first-suggested wide-range GO TENG humidity sensor with a high sensitivity of 500% (V/V-%RH) can be utilized in portable electronics, as well as industrial applications.

## Methods

### Chemicals and reagents

Graphite powder with particle size <45 μm was purchased from Sigma Aldrich. All other reagents including potassium permanganate (KMnO_4_), sulfuric acid (H_2_SO_4_), H_2_O_2_ (30 wt%), sodium nitrate (NaNO_3_) and hydrochloric acid (HCl) were bought from Chem Lab Co., Belgium. All chemicals were of analytical grade and used as received without further purification. The aqueous solutions were prepared using deionized water (DI, 18.2 MΩ, Millipore).

### Synthesis of graphene oxide

Graphene oxide was synthesized from natural graphite powder via a modified Hummers method as previously reported in the literature^44,45^. Briefly, 2 g of graphite powder was added to H_2_SO_4_ (12 mL, 98%) and then stirred for 1 h at 80 °C. Subsequently, 80 mL of H_2_SO_4_ was added to the mixture and the beaker was placed in an ice-bath followed by the addition of NaNO_3_ (2 g) while stirring for 10 min. Afterwards, 8 g of KMnO_4_ (4 wt %) was slowly added to the solution under vigorous stirring for additional 15 min. The green paste was then transferred to an oil bath (38–40 °C) followed by 90 min stirring. The resulted brownish solution was diluted with 160 mL of deionized (DI) water and heated at 95 °C for 30 min. Finally, the oxidation process was stopped by the addition of 400 mL DI water and H_2_O_2_ (16 mL, 30%). The orange-golden suspension was filtered and washed with diluted HCl and then DI water until the neutral pH (~5–6) was achieved. The filtered brown dough was redispersed in a certain amount of DI water and cleansed by centrifugation at 2500 rpm for 15 min and then 4000 rpm for 30 min to remove any un-exfoliated and tiny graphite sheets, respectively. Finally, GO nanosheets were prepared by sonication of the filtered graphite oxide suspension using an ultrasonic bath for 30 minutes.

### GO paper preparation

Large scale GO papers were prepared via a facile method. For this purpose, a plastic mold with desired dimensions was made. 20 mL of concentrated GO suspension (~7 mg/mL) was then poured into the mold and let it dry under ambient condition without any disturbance. Using this straightforward approach, one can prepare large scale GO papers with demanded sizes and control the thickness by adjusting the concentration as well as the volume of the GO suspension.

### Fabrication of GO TENG

Aluminum tape was used as the back-contact for GO paper as well as for the Kapton film to construct lower and upper electrodes, respectively. Two electrodes can be connected to the gauges of a tapping device (Fig. S5), by which can regulate the frequency and the force of tapping, as well as the spacing between two electrodes. The tests were performed under the vertical force of 8.3 N at frequencies from 1 up to 4 Hz. Electrodes had rectangular shape with the size of 8 × 8 cm^2^ and spacing of 2 cm between two electrodes. For humidity sensing, the size of electrodes reduced to 2 × 2 cm^2^ in order to enhance the accuracy of the GO TENG sensor.

### Humidity sensing tests

The ambient humidity was regulated by a typical humidifier. To report the amount of relative humidity (RH), a hygrometer was situated quite close to the surface of GO electrode. Humidity sensing tests were performed on the GO electrodes with the size of 8 × 8 cm^2^ and 2 × 2 cm^2^. The vertical tapping was applied using two approaches, utilizing a tapping device at the frequency of 2 Hz and the force of 8.3 N and also tapping by a human finger.

### Instruments and characterizations

TEM images were obtained with a Zeiss (EM10C-80KV) instrument. The morphology of the prepared GO nanosheets and also GO paper was studied by a field emission scanning electron microscope (MIRA3, Tescan). UV–Vis spectroscopy was performed on a Lambda25 (Perkin-Elmer, USA) spectrophotometer using a 1.0 cm quartz cell. The X-ray diffraction (XRD) pattern was recorded using a STOE (STADI P) instrument operating with Cu–Kα radiation (λ = 1.54178 A°) at 40 kV/30 mA. An Ivium Compactstat and an oscilloscope (DSO1022A) were used for current and voltage recording, respectively.

## Supplementary information

Supplementary Information.

Supplementary Information 2.

Supplementary Information 3.

## Data Availability

Derived data supporting the findings of this study are available from the corresponding author on request.

## References

[1] Wang ZL, Wu W (2012). Nanotechnology-enabled energy harvesting for self-powered micro-/nanosystems. Angew. Chem. Int. Ed..

[2] Wang L, Lin L, Wang ZL (2015). Triboelectric nanogenerators as self-powered active sensors. Nano Energy.

[3] Wang ZL, Song J (2006). Piezoelectric nanogenerators based on zinc oxide nanowire arrays. Science.

[4] Wang ZL (2013). Triboelectric nanogenerators as new energy technology for self-powered systems and as active mechanical and chemical sensors. ACS Nano.

[5] Yang Y (2012). Pyroelectric nanogenerators for harvesting thermoelectric energy. Nano Lett..

[6] Wang ZL (2014). Triboelectric nanogenerators as new energy technology and self-powered sensors – Principles, problems and perspectives. Faraday Discuss..

[7] Fan FR, Tang W, Wang ZL (2016). Flexible nanogenerators for energy harvesting and self-powered electronics. Adv. Mater..

[8] Song W (2016). Nanopillar arrayed triboelectric nanogenerator as a self-powered sensitive sensor for a sleep monitoring system. ACS Nano.

[9] Meng X (2018). Triboelectric nanogenerator as a highly sensitive self-powered sensor for driver behavior monitoring. Nano Energy.

[10] Yang J (2015). Eardrum-inspired active sensors for self-powered cardiovascular system characterization and throat-attached anti-interference voice recognition. Adv. Mater..

[11] Pu X (2017). Ultrastretchable, transparent triboelectric nanogenerator as electronic skin for biomechanical energy harvesting and tactile sensing. Sci. Adv..

[12] Tarelho JPG (2018). Graphene-based materials and structures for energy harvesting with fluids – A review. Mater. Today d.

[13] Zhao F, Cheng H, Zhang Z, Jiang L, Qu L (2015). Direct power generation from a graphene oxide film under moisture. Adv. Mater..

[14] Tian H (2013). Flexible electrostatic nanogenerator using graphene oxide film. Nanoscale.

[15] Guo H (2017). Self-sterilized flexible single-electrode triboelectric nanogenerator for energy harvesting and dynamic force sensing. ACS Nano.

[16] Valentini L, Rescignano N, Puglia D, Cardinali M, Kenny J (2015). Preparation of alginate/graphene oxide hybrid films and their integration in triboelectric generators. Eur. J. Inorg. Chem..

[17] Harnchana V (2018). Enhanced power output of a triboelectric nanogenerator using polydimethylsiloxane modified with graphene oxide and sodium dodecyl sulfate. ACS Appl. Mater. Interfaces.

[18] Toda K, Furue R, Hayami S (2015). Recent progress in applications of graphene oxide for gas sensing: A review. Analytica Chimica Acta.

[19] Cai J, Lv C, Aoyagi E, Ogawa S, Watanabe A (2018). Laser direct writing of a high-performance all-graphene humidity sensor working in a novel sensing mode for portable electronics. ACS Appl. Mater. Interfaces.

[20] Lv C (2019). Recent advances in graphene-based humidity sensors. Nanomaterials.

[21] Bi H (2013). Ultrahigh humidity sensitivity of graphene oxide. Sci. Rep..

[22] Borini S (2013). Ultrafast graphene oxide humidity sensors. ACS Nano.

[23] Naik G, Krishnaswamy S (2016). Room-temperature humidity sensing using graphene oxide thin films. Graphene.

[24] Park EU (2018). Correlation between the sensitivity and the hysteresis of humidity sensors based on graphene oxides. Sens. Actuat. B Chem..

[25] Wan N (2018). Microstructure related synergic sensoring mechanism in graphene oxide humidity sensor. J. Phys. Chem. C.

[26] Wee B-H, Khoh W-H, Sarker AK, Lee C-H, Hong J-D (2015). A High-Performance Moisture Sensor Based on Ultralarge Graphene Oxide. Nanoscale.

[27] Zhang D, Tong J, Xia B, Xue Q (2014). Ultrahigh performance humidity sensor based on layer-by-layer self-assembly of graphene oxide/polyelectrolyte nanocomposite film. Sens. Actuat. B Chem..

[28] Leng X, Luo D, Xu Z, Wang F (2018). Modified graphene oxide/nafion composite humidity sensor and its linear response to the relative humidity. Sens. Actuat. B Chem..

[29] Xu J, Gu S, Lu B (2015). Graphene and graphene oxide double decorated SnO_2_ nanofibers with enhanced humidity sensing performance. RSC Adv..

[30] Zhang D, Liu J, Xia B (2016). Layer-by-layer self-assembly of zinc oxide/graphene oxide hybrid toward ultrasensitive humidity sensing. IEEE Electron Device Lett..

[31] Sun L, Haidry AA, Fatima Q, Li Z, Yao Z (2018). Improving the humidity sensing below 30% RH of TiO_2_ with GO modification. Mater. Res. Bull..

[32] Burman D, Ghosh R, Santra S, Guha PK (2016). Highly proton conducting MoS_2_/graphene oxide nanocomposite based chemoresistive humidity sensor. RSC Adv..

[33] Jha RK, Burman D, Santra S, Guha PK (2017). WS_2_/GO nanohybrids for enhanced relative humidity sensing at room temperature. IEEE Sens. J..

[34] Guo H (2014). Airflow-induced triboelectric nanogenerator as a self-powered sensor for detecting humidity and airflow rate. ACS Appl. Mater. Interfaces.

[35] Su Y (2017). Novel high-performance self-powered humidity detection enabled by triboelectric effect. Sens. Actuat. B Chem..

[36] Ren Z (2019). Environmental energy harvesting adapting to different weather conditions and self-powered vapor sensor based on humidity-responsive triboelectric nanogenerators. ACS Appl. Mater. Interfaces.

[37] Nguyen V, Yang R (2013). Effect of humidity and pressure on the triboelectric nanogenerator. Nano Energy.

[38] Zhao Z (2016). Freestanding flag-type triboelectric nanogenerator for harvesting high-altitude wind energy from arbitrary directions. ACS Nano.

[39] Jao Y (2018). A textile-based triboelectric nanogenerator with humidity-resistant output characteristic and its applications in self-powered healthcare sensors. Nano Energy.

[40] Ray TR (2019). Bio-integrated wearable systems: A comprehensive review. Chem. Rev..

[41] Mohammadpour R (2017). Flexible triboelectric nanogenerator based on high surface area TiO_2_ nanotube arrays. Adv. Eng. Mater..

[42] Li N, Chen X, Chen X, Ding X, Zhao X (2017). Ultrahigh humidity sensitivity of graphene oxide combined with Ag nanoparticles. RSC Adv..

[43] Huang YX, Cheng HH, Shi GQ, Qu LT (2017). Highly efficient moisture-triggered nanogenerator based on graphene quantum dots. ACS Appl. Mater. Interfaces.

[44] Esfandiar A, Akhavan O, Irajizad A (2011). Melatonin as a powerful bio-antioxidant for reduction of graphene oxide. J. Mater. Chem..

[45] Asadian E, Shahrokhian S, Zad AI, Ghorbani-Bidkorbeh F (2017). Glassy carbon electrode modified with 3D graphene-carbon nanotube network for sensitive electrochemical determination of methotrexate. Sens. Actuator B Chem..

