# Supplementary material for: Graphene Oxide Papers in Nanogenerators for Self-Powered Humidity Sensing by Finger Tapping
Source: Sci Rep. 2020 Apr 30;10:7312. doi: 10.1038/s41598-020-64490-7 (PMC7192944; doi:10.1038/s41598-020-64490-7)
Supplement: Supplementary file 1 — Supplementary Information. [file 41598_2020_64490_MOESM1_ESM.pdf]

Graphene Oxide Papers in Nanogenerators for Self-Powered Humidity Sensing by Finger Tapping

Faezeh Ejehi, Raheleh Mohammadpour, Elham Asadian, Pezhman Sasanpour, Somayeh Fardindoost, Omid Akhavan

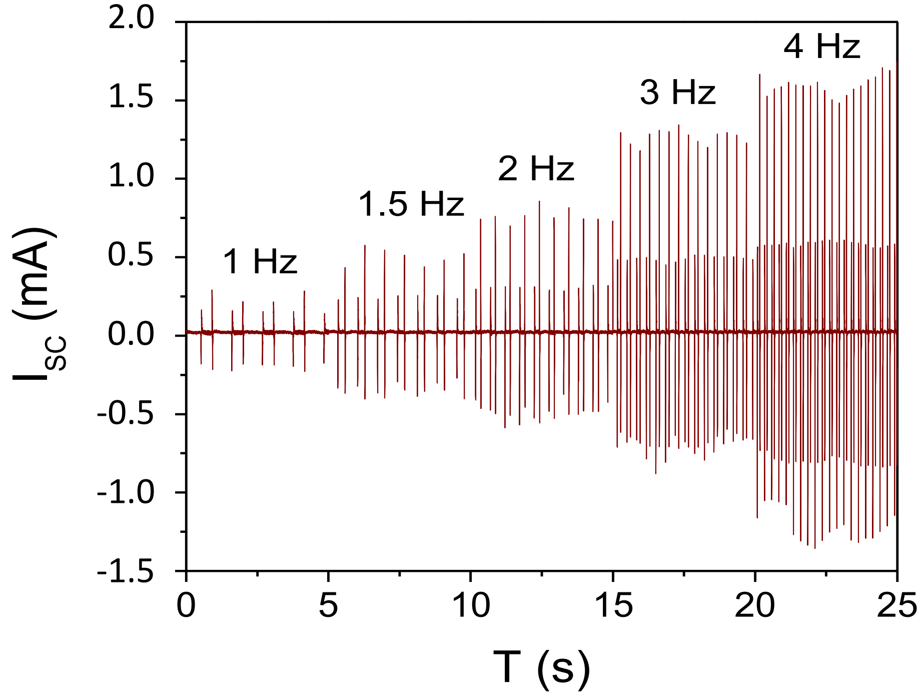

Figure S1. Amplified current output at frequencies of 1 Hz to 4 Hz.

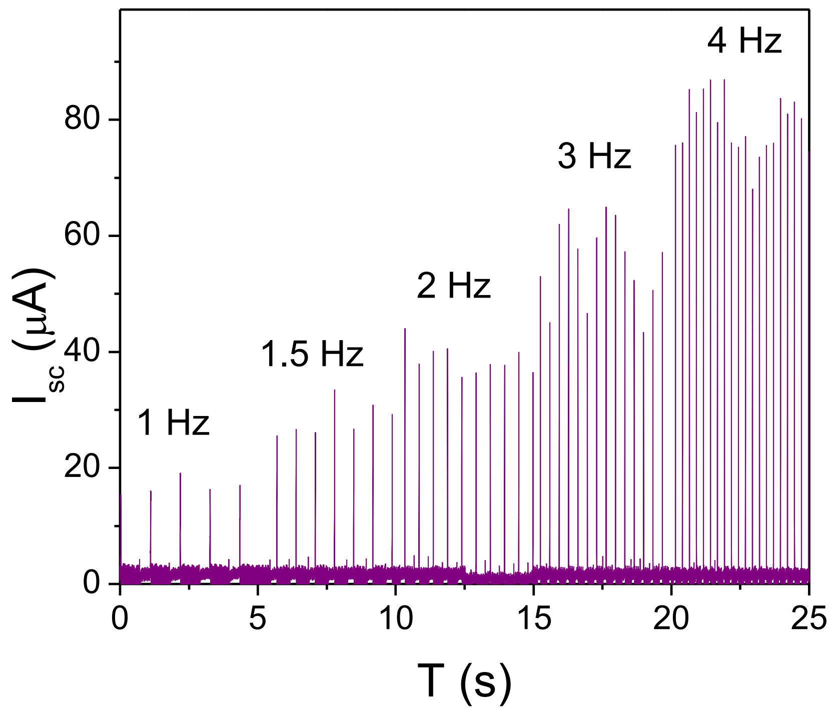

Figure S2. Rectified current output at frequencies of 1 Hz to 4 Hz.

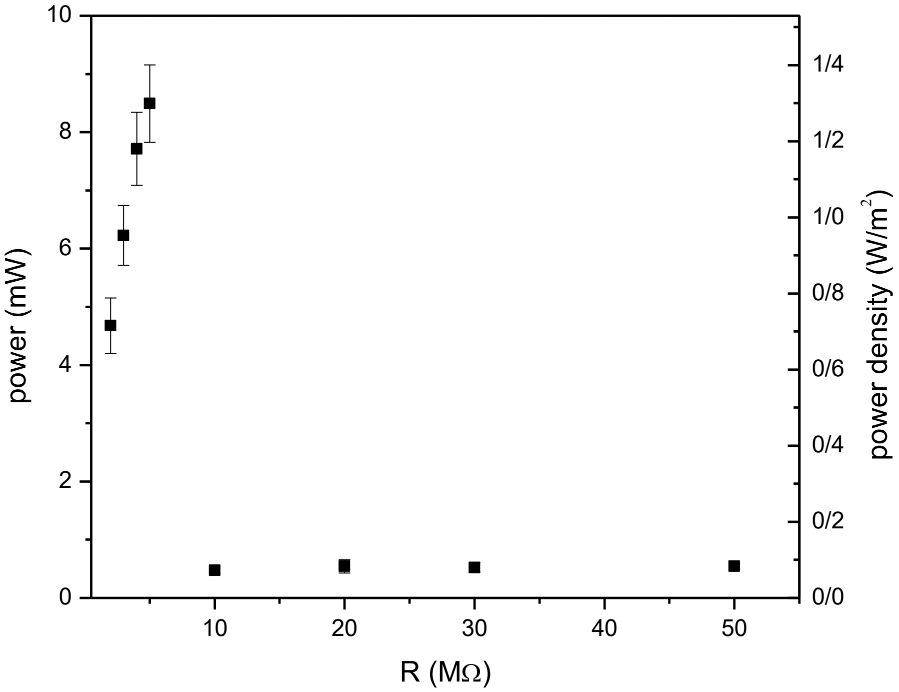

Figure S3. Dependence of the generated power of the TENG on the external loading resistance.

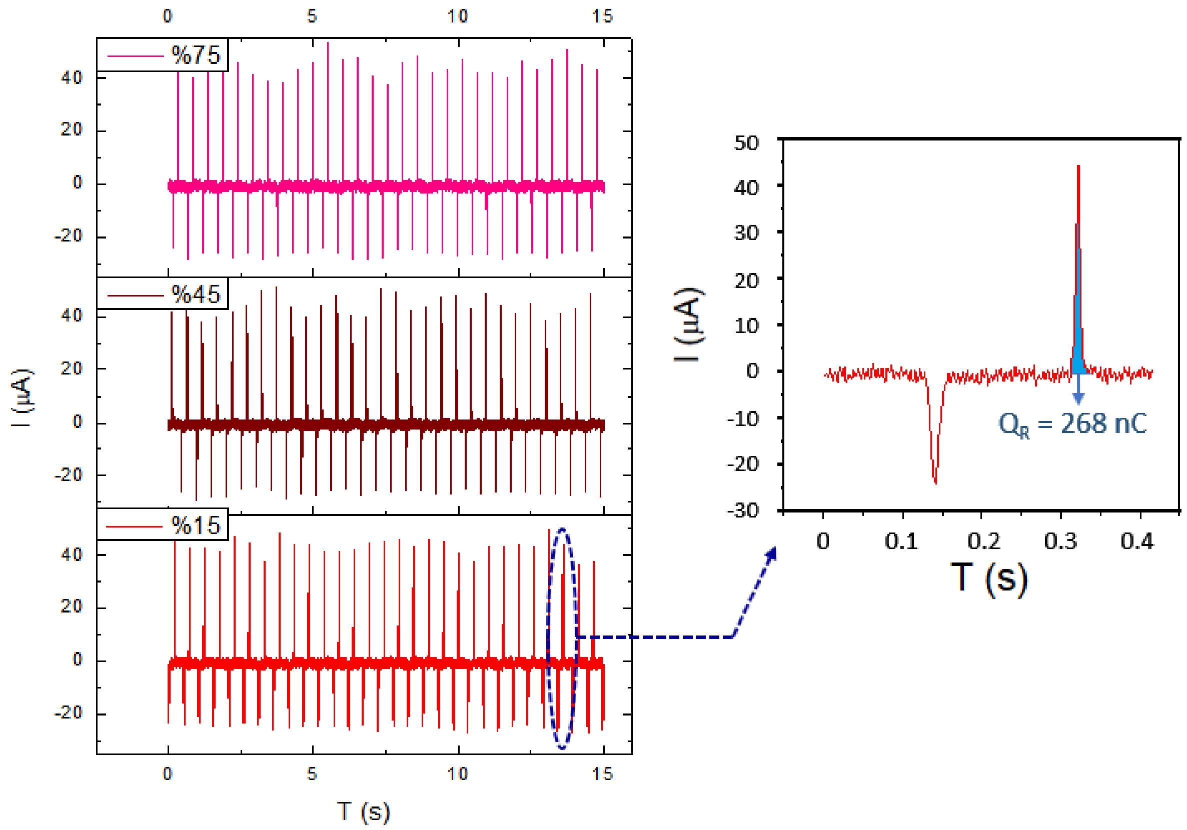

Figure S4. Trivial variation of the output current at various amount of RH for the electrode with surface area of 64 cm^2.

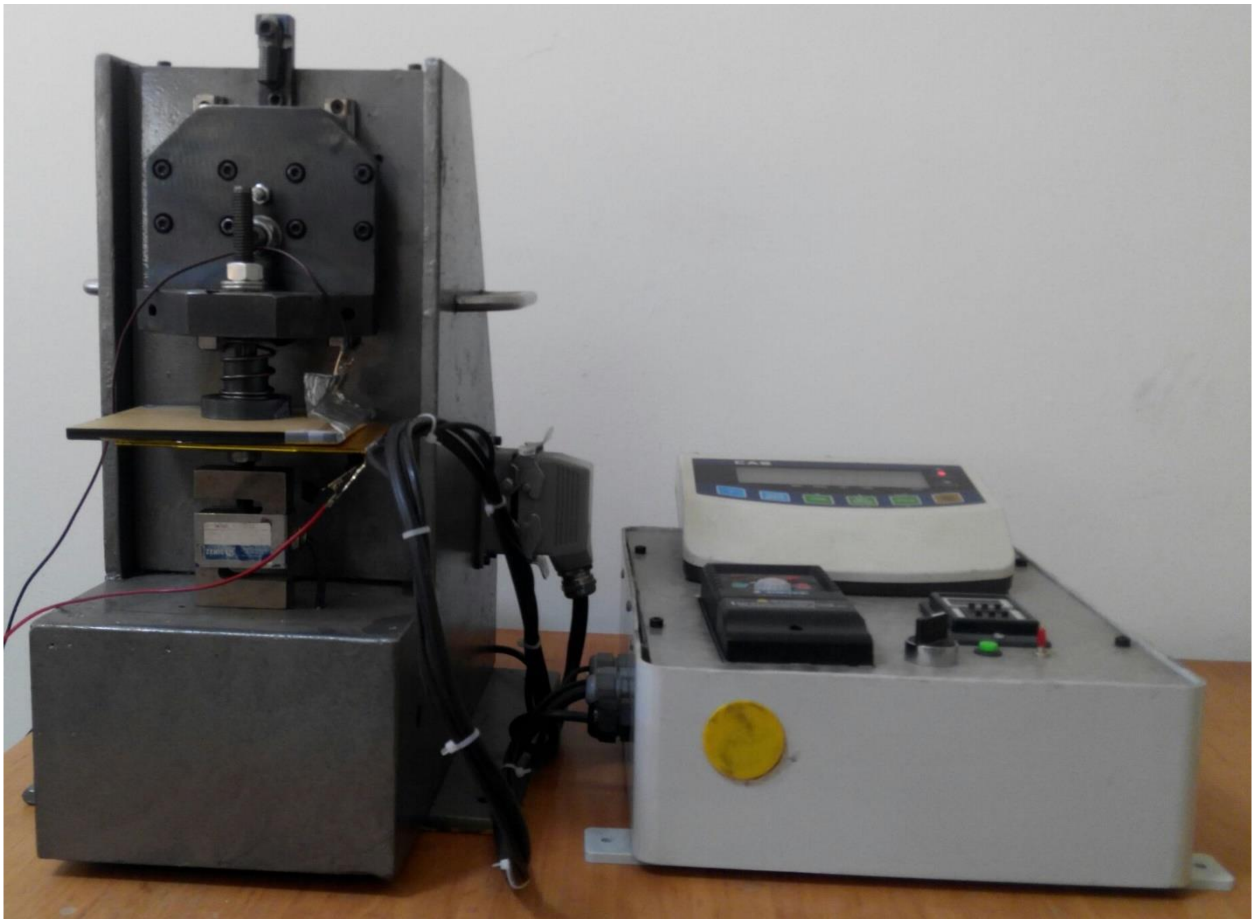

Figure S5. Digital photograph of the tapping device.

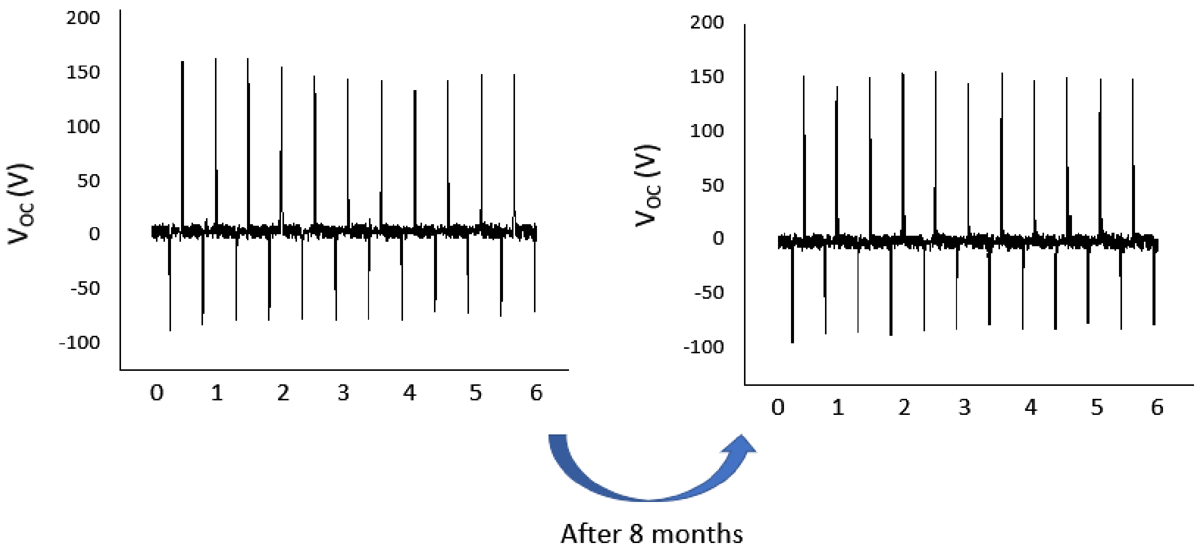

Figure S6. The generated voltage value under tapping at 2 Hz in RH=20%, before (left) and after (right) 8 months, in order to confirm the long-term stability of the GO paper electrode.
